# Redistribution of Extracellular Superoxide Dismutase Causes Neonatal Pulmonary Vascular Remodeling and PH but Protects Against Experimental Bronchopulmonary Dysplasia

**DOI:** 10.3390/antiox7030042

**Published:** 2018-03-14

**Authors:** Laurie G. Sherlock, Ashley Trumpie, Laura Hernandez-Lagunas, Sarah McKenna, Susan Fisher, Russell Bowler, Clyde J. Wright, Cassidy Delaney, Eva Nozik-Grayck

**Affiliations:** 1 Department of Pediatrics, Section of Neonatology, University of Colorado Anschutz Medical Campus, Aurora, CO 80045, USA; laura.sherlock@ucdenver.edu (L.G.S.); sarah.mckenna@ucdenver.edu (S.M.); susan.fisher@ucdenver.edu (S.F.); clyde.wright@ucdenver.edu (C.J.W.); cassidy.delaney@childrenscolorado.org (C.D.); 2 Cardiovascular Pulmonary Research Laboratories, University of Colorado Anschutz Medical Campus, Aurora, CO 80045, USA; Ashley.trumpie@ucdenver.edu (A.T.); ana-laura.hernandez@ucdenver.edu (L.H.-L.); 3 Department of Medicine, National Jewish Health, Denver, CO 80206, USA; bowlerr@njhealth.org; 4 Pediatric Critical Care Medicine, University of Colorado Anschutz Medical Campus, Aurora, CO 80045, USA

**Keywords:** extracellular superoxide dismutase, R213G single nucleotide polymorphism, neonate, bronchopulmonary dysplasia, pulmonary hypertension

## Abstract

Background: A naturally occurring single nucleotide polymorphism (SNP), (R_213_G), in extracellular superoxide dismutase (SOD3), decreases SOD3 matrix binding affinity. Humans and mature mice expressing the R_213_G SNP exhibit increased cardiovascular disease but decreased lung disease. The impact of this SNP on the neonatal lung at baseline or with injury is unknown. Methods: Wild type and homozygous R_213_G mice were injected with intraperitoneal bleomycin or phosphate buffered saline (PBS) three times weekly for three weeks and tissue harvested at 22 days of life. Vascular and alveolar development were evaluated by morphometric analysis and immunostaining of lung sections. Pulmonary hypertension (PH) was assessed by right ventricular hypertrophy (RVH). Lung protein expression for superoxide dismutase (SOD) isoforms, catalase, vascular endothelial growth factor receptor 2 (VEGFR2), endothelial nitric oxide synthase (eNOS) and guanosine triphosphate cyclohydrolase-1 (GTPCH-1) was evaluated by western blot. SOD activity and SOD3 expression were measured in serum. Results: In R_213_G mice, SOD3 lung protein expression decreased, serum SOD3 protein expression and SOD serum activity increased compared to wild type (WT) mice. Under control conditions, R_213_G mice developed pulmonary vascular remodeling (decreased vessel density and increased medial wall thickness) and PH; alveolar development was similar between strains. After bleomycin injury, in contrast to WT, R_213_G mice were protected from impaired alveolar development and their vascular abnormalities and PH did not worsen. Bleomycin decreased VEGFR2 and GTPCH-1 only in WT mice. Conclusion: R_213_G neonatal mice demonstrate impaired vascular development and PH at baseline without alveolar simplification, yet are protected from bleomycin induced lung injury and worsening of pulmonary vascular remodeling and PH. These results show that vessel bound SOD3 is essential in normal pulmonary vascular development, and increased serum SOD3 expression and SOD activity prevent lung injury in experimental bronchopulmonary dysplasia (BPD) and PH.

## 1. Introduction

Bronchopulmonary dysplasia (BPD) is a disorder of lung development affecting preterm infants resulting in respiratory morbidities that can persist through adulthood [1]. Despite advances in neonatal care, the incidence of BPD is increasing [2]. A subset of infants with BPD also develop pulmonary hypertension (PH), characterized by impaired angiogenesis, pulmonary vascular remodeling and right ventricular failure, which significantly increases the morbidity and mortality [3,4]. Interventions to prevent and treat BPD complicated by PH are limited, thus there is an urgent need to improve understanding of the mechanisms contributing to the development of these frequent and burdensome diseases of prematurity. 

Oxidative stress is central to the etiology of BPD and PH [5,6]. Antioxidant enzymes and essential nutrients that contribute to antioxidant status increase prior to birth and prepare infants for the increased partial pressure of oxygen with post-natal room air breathing [6]. Premature neonates have decreased antioxidant defenses, and increased oxidative stress due to oxygen therapy, infection, and mechanical ventilation [6]. However, despite compelling evidence that an imbalance of oxidative stress contributes to BPD and PH, generalized antioxidant therapies have not proven efficacious to preventing or treating these diseases [6]. An enhanced understanding of how antioxidants impact regional redox signaling may lead to more effective targeted antioxidant therapies that improve clinical outcomes. 

A human single nucleotide polymorphism (SNP) of the antioxidant enzyme extracellular superoxide dismutase (EC-SOD or SOD3) provides insight into how the specific location of SOD3 influences human disease risk. The R_213_G SNP (rs1799895) is observed in 3–6% of studied populations; this SNP does not change enzyme activity, but lowers tissue binding [7]. SOD3 is typically tethered to the extracellular matrix (ECM) by a series of positively charged amino acids that bind to the negatively charged ligands in the ECM and glycocalyx of endothelial and epithelial cells [8,9]. The rs1799895 SNP leads to a single amino acid substitution from arginine to glycine at position 213 within the matrix binding region, lowering the matrix binding affinity of SOD3, and redistributing SOD3 from the lung parenchyma and vasculature into the plasma and epithelial lining fluid [7,10,11]. In humans, the R_213_G SNP is associated with decreased risk of chronic obstructive pulmonary disease (COPD) and asthma [12,13]. Adult mice harboring the same SNP are protected from intratracheal lipopolysaccharide (LPS) inflammation, bleomycin induced pulmonary fibrosis and PH, and ovalbumin (OVA) induced airway obstruction [7,12,14]. Conversely, this SNP is associated with worse outcomes in ischemic heart disease [15], and adult mice exhibit PH at baseline and exaggerated chronic hypoxia-induced PH [7,14]. The divergent risk profiles imparted by the R_213_G SNP in different diseases illustrates the importance of the site of oxidative stress and antioxidant defenses in disease pathogenesis.

SOD3 is developmentally regulated, and SOD3 deficient neonatal mice exhibit impaired alveolar and pulmonary vascular development at baseline [16]. The expression and activity of SOD3 is decreased in neonatal models of BPD and/or PH [17,18]. Overall SOD3 content influences response to disease, as overexpression of SOD3 ameliorates experimental BPD and/or PH [19,20,21], and SOD3 deficiency aggravates injury in experimental BPD and PH [16]. These findings provide evidence that SOD3 content is important in the normal developing lung and with neonatal lung injury, however the impact of the distribution of SOD3 on the developing lung and with injury is unknown. The R_213_G SNP provides a unique opportunity to interrogate the impact of SOD3 distribution as it only changes SOD3 location. We hypothesized that the redistribution of SOD3 from the lung and vasculature into the extracellular fluids due to the R_213_G SNP would impair baseline alveolar and/or vascular development and worsen neonatal BPD and PH.

## 2. Methods

### 2.1. Mouse Model

All animal studies were approved by the University of Colorado Denver Institutional Animal Care and Use Committee (IACUC) (B-7117(01)1E). C57BL/6 wild-type mice (Jackson Laboratory, Bar Harbor, ME, USA) and genetically engineered mice on the C57BL/6 background with knock-in of the human R_213_G SNP into the matrix binding region were raised in Denver altitude [7]. Bleomycin, a chemotherapeutic agent that induces significant lung fibrosis and inflammation in adults [22,23], has been adapted into a model of experimental BPD and PH in neonatal rodents, characterized by alveolar simplification, decreased vascular development, and vascular remodeling similar to infants with BPD complicated by PH [16,24,25,26]. Neonatal male and female mice were injected with 10 μL of intraperitoneal phosphate buffered saline (PBS) or bleomycin (3 units/kg/dose, dissolved in 10 μL PBS) (Hospira, Lake Forest, IL, USA), three times a week for three weeks for a total bleomycin exposure of 27 units/kg. Bleomycin was dosed per weight at each injection. Injections were started on day of life 2 and ended on day of life 22. Since the lungs of newborn mice are in the saccular stage of lung development, the 2-day old mouse at the onset of treatment models the stage of lung development of human infants born at 28–32 weeks [27]. Mice were anesthetized with 1.5% isoflurane, then euthanized with 100 μL of Fatal-Plus solution (Vortech Pharmaceuticals, Dearbon, MI, USA) and thoracotomy for tissue harvesting at 22 days of life, allowing for assessment during the late alveolar stage of development. Lungs were flushed with PBS and were either frozen in All-Protect (Qiagen, Valencia, CA, USA) for protein isolation, or inflation-fixed at 25 cm H_2_O with 4% paraformaldehyde for paraffin embedding.

### 2.2. Immunohistochemistry

Immunohistochemistry for alpha smooth muscle actin (α-SMA)was performed as previously described with mouse monoclonal α-SMA antibody (1:1500, clone1A4; Sigma, St. Louis, MO, USA) and Mouse on Mouse (M.O.M.) immunodetection kit with ready-to-use anti-mouse secondary IgG antibody (Vector Laboratories, Burlingame, CA, USA) [16]. Additionally, lung sections were stained with rabbit anti-vWF (1:1500, Sigma-Aldrich, St. Louis, MO, USA), and ready-to- use horse radish peroxidase (HRP) conjugated anti-rabbit IgG (ImmPress Kit, Vector Laboratories, Burlingame, CA, USA). Slides were developed with Vector very intense purple (VIP) peroxidase (HRP) substrate kit (Vector Laboratories, Burlingame, CA, USA) and counterstained with methyl green.

### 2.3. Evaluation of Pulmonary Vascular Structure

Vessel density was assessed by counting the number of vessels <30 μm staining positive for von Willebrand Factor (vWF) per high-power field (20×). Lung fields containing large vessels or airways were excluded, and a minimum of 8 fields were included per mouse. Medial wall thickness was calculated for α-SMA stained vessels 30–100 μm located adjacent to airways [28]. The width of the medial wall was measured in four perpendicular locations and an average was calculated. The external diameter was measured in two locations, this was then averaged and divided by two to calculate the average radius. The medial wall thickness (MWT) was expressed as average width of the medial wall/average radius. The analysis was performed by an investigator blinded to the experiment group.

### 2.4. Evaluation of Alveolar Development

Radial alveolar counts (RACs) were calculated on hematoxylin and eosin stained lung sections by identifying the terminal bronchiole, drawing a perpendicular line to the lung periphery, and counting each intersection of lung alveoli [29]. At least five images were processed per mouse at 10× magnification. Mean linear intercept (MLI) was measured using Metamorph Basic (Molecular Devices Sunnyvale, San Jose, CA, USA). At least 7 non-overlapping sections per mouse were assessed at 20× magnification. Fields with large airways or vessels were excluded. The analysis was performed by an investigator blinded to the experiment group.

### 2.5. Evaluation of Right Ventricular Hypertrophy

Fulton’s index, defined as the ratio of right ventricle (RV) weight divided by left ventricle (LV) plus septum (S) (RV/(LV + S)), was determined as a measure of right ventricular hypertrophy. Hearts were stored in 4% paraformaldehyde until dissected, as previously described [16].

### 2.6. Protein Expression

Western blots were performed on 25 μg protein from total lung homogenates prepared in tissue protein extraction reagent (TPER) lysis buffer with protease and phosphatase inhibitors or 2 μL serum as previously described [30]. Blots were blocked for 1 h using 5% nonfat milk in 1× TBST, then probed overnight at 4 °C using the following antibodies: catalase (1:500, Abcam, Cambridge, UK), superoxide dismutase 1 (SOD1) (1:1000, Abcam, Cambridge, UK), superoxide dismutase 2 (SOD2) (1:1000, Millipore, Billerica, MA, USA), SOD3 (1:1000, Santa Cruz Biotechnology, Santa Cruz, CA, USA), vascular endothelial growth factor receptor 2 (VEGFR2) (1:500, Cell Signaling, Danvers, MA, USA), endothelial nitric oxide synthase (eNOS) (1:1000, BD Biosciences, San Jose, CA, USA), guanosine triphosphate cyclohydrolase-1 (GTPCH-1) (1:1000, Abcam, Cambridge, UK). After washing, blots were incubated for 1 hour at room temperature with the species-appropriate secondary IgG antibody (1:10,000, Millipore, Billerica, MA, USA). They were then developed with SuperSignal Femto (ThermoScientific) or Western Lightning ECL. Blots were then stripped and reprobed with β-actin mouse monoclonal antibody (1:10,000, Sigma-Aldrich, St. Louis, MO, USA) as a loading control. Images were obtained on FluorChemM camera system. Densitometry was done using ImageJ (National Institute of Health, Bethesda, MD, USA) and subtracting for background. 

### 2.7. SOD Activity Assay

SOD activity was measured on serum as previously described using a SOD activity assay kit (Dojindo Molecular Technologies, Santa Clara, CA, USA). Grossly hemolyzed samples were excluded to avoid elevated SOD activity resulting from the release of red blood cell SOD1. The standard curve was performed using bovine erythrocyte SOD1 (Sigma Aldrich, St Louis, MO, USA). SOD activity data were expressed as units of SOD activity per ml of serum [14].

### 2.8. Statistical Analysis

Data were analyzed using Prism (GraphPad Software, La Jolla, CA, USA) by unpaired *t*-test or two-way analysis of variance (ANOVA). Post-hoc analysis was performed using Tukey’s test when significant differences were found between groups. Data are expressed as mean ± SD. Significance was defined as *p* < 0.05.

## 3. Results

### 3.1. The R_213_G SNP Redistributed SOD3 from The Lung to The Extracellular Fluid in Neonatal Mice

**Figure 1:**
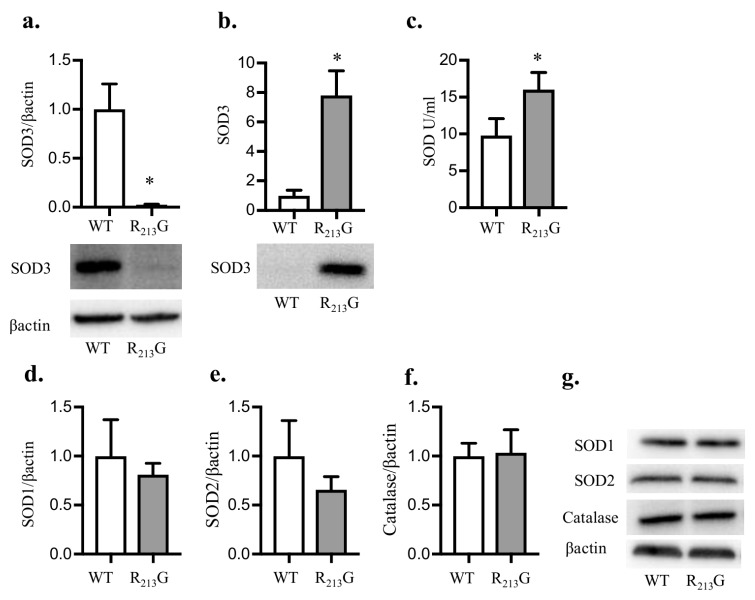
The R_213_G single nucleotide polymorphism (SNP) redistributed superoxide dismutase (SOD3) from the lung into the plasma at 22 days. SOD3 protein content in the lung and serum was evaluated by western blot analysis in control WT and R_213_G mice at 22 days of age. A quantity of 25 μg lung protein or 2 μL serum was loaded onto the gels. (**a**) Lung SOD3 (**b**) Serum SOD3. SOD activity in serum was measured using the SOD activity assay (Dojindo). Representative blots are shown below optical density normalized to β-actin and expressed relative to WT mice in Figure 1a,b. (**c**) Serum SOD Activity (units per mL). Several other key antioxidant enzymes were evaluated in the lung by western blot analysis. Representative blots are shown along with optical density normalized to β-actin and expressed relative to WT mice (**d**) Lung superoxide dismutase 1 (SOD1) (**e**) Lung superoxide dismutase 2 (SOD2) (**f**) Lung Catalase. (**g**) Representative blots for SOD1, SOD2, catalase and β-actin are shown).* *p* < 0.05 vs. WT PBS by unpaired *t*-test, *n* = 5–6 for all groups. WT: wild type.

We first tested how the R_213_G SNP impacts SOD3 distribution in the immature lung. At 22 days, we observed significantly less SOD3 protein in the lungs of R_213_G mice (Figure 1a) and elevated serum SOD3 compared to wild type (WT) mice (Figure 1b), recapitulating what we reported previously in adult mice [7]. Serum SOD activity was also elevated in the R_213_G mice compared to WT (Figure 1c). We next evaluated if the R_213_G mice compensate for low lung SOD3 content by upregulating other key antioxidant enzymes in the neonatal lung. There was no change in lung SOD1 (Figure 1d,g), SOD2 (Figure 1e,g), or catalase (Figure 1f,g) at 22 days in mice expressing the SOD3 R_213_G SNP compared to WT mice.

### 3.2. Mice Expressing the R_213_G SNP Demonstrated Impaired Pulmonary Vascular Development and PH Under Control Conditions

**Figure 2:**
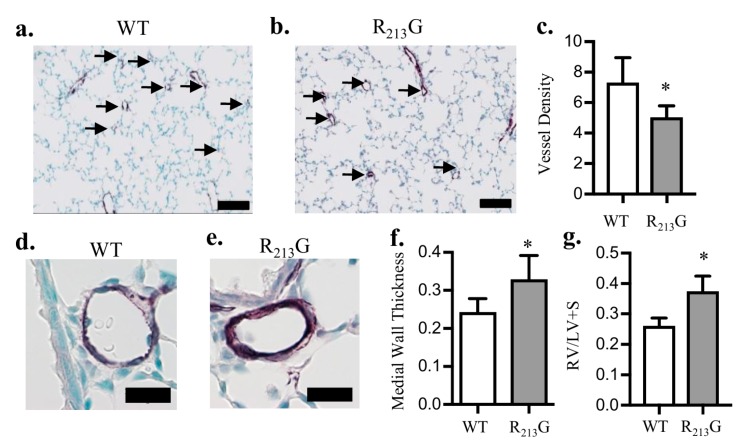
Mice expressing the R_213_G SNP had impaired pulmonary vascular development and pulmonary hypertension at 22 days. (**a**,**b**) Representative images of von willebrand factor (vWF) (purple) staining in (**a**) WT and (**b**) R_213_G control mice. Arrows indicate vessels < 30 μm, scale bar = 100 μm. (**c**) Vessel density in WT and R_213_G mice, * *p* < 0.05 by unpaired *t*-test, *n*= 5–6. (**d**,**e**) Representative images of alpha smooth muscle actin (αSMA) (purple) staining in (**d**) WT and (**e**) R_213_G mice, scale bar = 20 μm. (**f**) Medial wall thickness in WT and R_213_G mice; * *p* < 0.05 vs. WT phosphate buffered saline (PBS) by unpaired *t*-test, *n* = 5–6. (**g**) Right ventricular (RV)/left ventricular (LV) + septal (S) (RV/LV + S) weights in WT and R_213_G control mice, * *p* < 0.05 vs. WT PBS by unpaired *t*-test, *n* = 5–6.

In order to evaluate the redistribution of SOD3 on vascular development, we analyzed pulmonary vascular density and remodeling at 22 days. Vessel density (<30 μm) was decreased in the R_213_G mice (Figure 2c). Medial wall thickness (MWT) in vessels 30–150 μm, a marker of vascular remodeling, was increased in R_213_G mice (Figure 2f). As a surrogate for PH, we measured right ventricular hypertrophy (RVH) by Fulton’s index and found RVH increased in mice expressing the R_213_G SNP compared to WT mice (Figure 2g). * *p* < 0.05 by unpaired *t*-test, *n* = 5–6.

### 3.3. Alveolar Development Was Not Altered in Mice Expressing the R_213_G SNP

**Figure 3:**
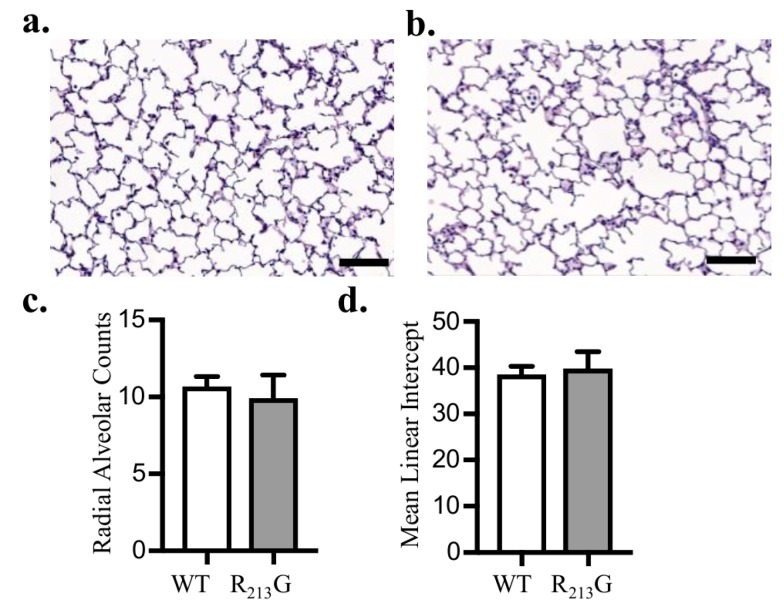
Mice expressing the R_213_G SNP exhibited normal alveolar development. Representative images of H & E stained lung in 22-day old (**a**) WT and (**b**) R_213_G control mice, scale bar = 100 μm. (**c**) Radial alveolar counts in control WT and R_213_G mice *p* > 0.05 by unpaired *t*-test, *n* = 7–8 (**d**) Mean linear intercepts in control WT and R_213_G mice. *p* > 0.05 vs. WT PBS by unpaired *t*-test, *n* = 6–8.

To evaluate the impact of the R_213_G distribution on alveolar development, we performed morphometric assessment of alveolarization at 22 days with radial alveolar counts (RAC) and mean linear intercept (MLI). RAC and MLI were similar between R_213_G and WT control mice (Figure 3c,d).

### 3.4. Bleomycin Did Not Alter Antioxidant Enzyme Expression in the Lung or Plasma in Either Strain

**Figure 4:**
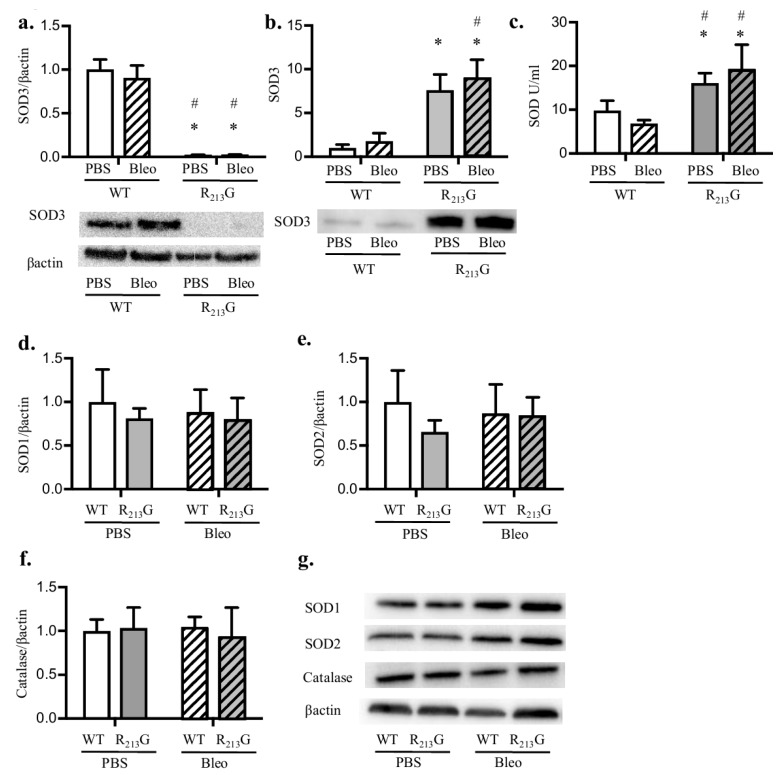
Bleomycin does not change R_213_G redistribution of SOD3. SOD3 protein content in the lung and serum was evaluated by western blot analysis in WT and R_213_G mice at 22 days of age after exposure to PBS (10 μL for 9 doses) or bleomycin (3 units/kg/dose dissolved in 10 μL of PBS for 9 doses). 25 μg lung protein or 2 μL serum was loaded onto the gels. (**a**) Lung SOD3 (**b**) Serum SOD3. Representative blots for Figure 4a,b are shown along with optical density normalized to β-actin and expressed relative to WT control mice. SOD activity was measured in serum. (**c**) Serum SOD Activity (units per ml). Several other key antioxidant enzymes were evaluated in the lung by western blot analysis. Representative blots are shown along with optical density normalized to β-actin and expressed relative to WT mice (**d**) Lung SOD1 (**e**) Lung SOD2 (**f**) Lung catalase. (**g**) Representative blots for SOD1, SOD2, catalase and β-actin. * *p* < 0.05 vs. WT PBS, # *p* < 0.05 vs. WT Bleo, all analysis by two-way ANOVA, *n* = 5–6 for all groups.

We next sought to determine how the R_213_G SNP impacts the response to bleomycin induced BPD and PH. First, we tested if bleomycin altered the content or distribution of SOD3 in the R_213_G mice by comparing bleomycin treated WT and R_213_G mice to the cohort of mice shown in Figure 1, Figure 2 and Figure 3. By western blot analysis, bleomycin did not change lung or serum protein SOD3 levels in either mouse strain (Figure 4a,b). Bleomycin additionally did not impact the R_213_G redistribution of SOD3. Bleomycin did not change serum SOD activity in either strain compared to controls (Figure 4c). When evaluating the lung for changes in key antioxidant enzymes, we found no change in SOD1 (Figure 4d,g), SOD2 (Figure 4e,g), or catalase (Figure 4f,g) in the lungs of either strain after bleomycin exposure.

### 3.5. Mice Expressing the R_213_G SNP Do Not Have Further Worsening of Pulmonary Vascular Development or PH after Bleomycin Injury

**Figure 5:**
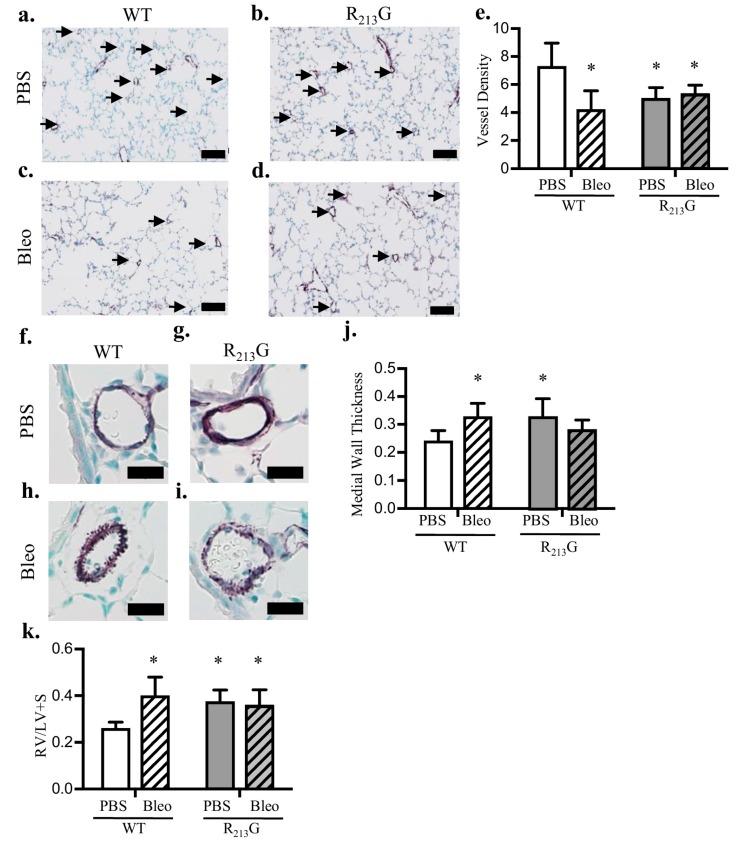
Bleomycin did not further impair the baseline pulmonary vascular impairment or worsen PH in the R_213_G mice. (**a**–**d**) Representative images of vWF (purple) staining in (**a**) WT PBS, (**b**) R_213_G PBS, (**c**) WT Bleo, (**d**) R_213_G Bleo. Arrows indicate vessels < 30 μm, scale bar = 100 μm. (**e**) Vessel density in WT and R_213_G mice following exposure to intraperitoneal (IP) PBS or bleomycin, * *p* < 0.05 vs. WT PBS by two-way ANOVA, *n* = 6–9. (**f**–**i**) Representative images of αSMA (purple) staining in (**f**) WT PBS, (**g**) R_213_G PBS, (**h**) WT Bleo, (**i**) R_213_G Bleo, scale bar = 20 μm. (**j**) Medial wall thickness in WT and R_213_G mice following IP PBS or bleomycin exposure, * *p* < 0.05 vs. WT PBS by two-way ANOVA, *n* = 6–7. (**k**) RV/LV + S weights in WT and R_213_G mice following IP PBS or bleomycin exposure, * *p* < 0.05 vs. WT PBS by two-way ANOVA, *n* = 5–8.

Intraperitoneal bleomycin is used as a rodent model of BPD and PH. We tested if the redistribution of SOD3 due to the R_213_G polymorphism would worsen bleomycin induced pulmonary vascular remodeling and PH. Recapitulating our previous findings, in WT mice, bleomycin resulted in pulmonary vascular remodeling with decreased pulmonary vessel density (Figure 5e), and increased medial wall thickness (Figure 5j). Bleomycin also induced PH in WT mice, shown by RVH (Figure 5k). In contrast, bleomycin had no effect on vessel density, MWT, or RVH in the R_213_G mice compared to baseline (Figure 5e,j,k).

### 3.6. The R_213_G SNP Mitigated Bleomycin Induced Alveolar Simplification at 22 Days

**Figure 6:**
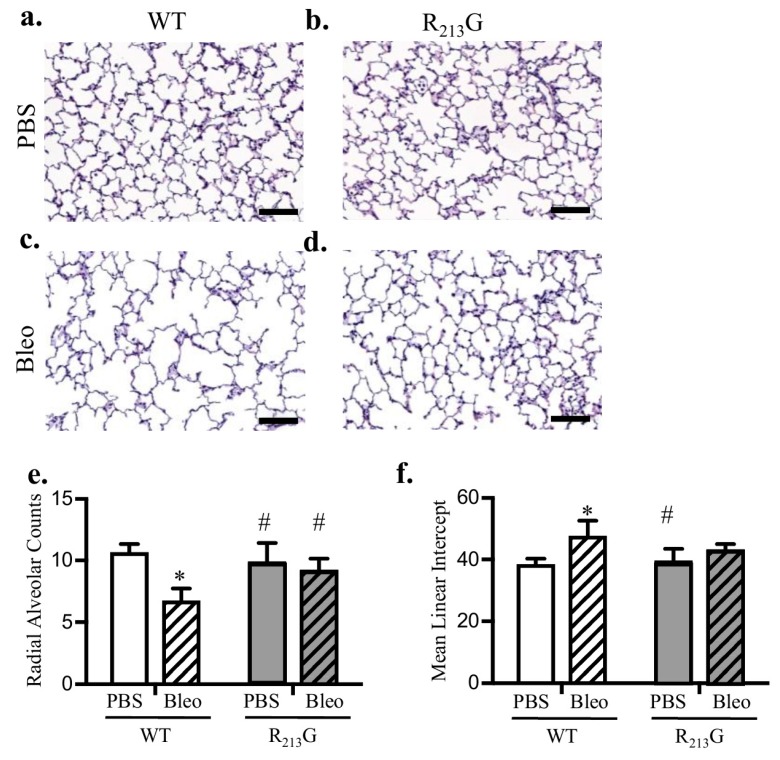
The SOD3 R_213_G SNP mitigated bleomycin induced alveolar simplification at 22 days. (**a**–**d**) Representative images of H&E staining in (**a**) WT PBS, (**b**) R_213_G PBS, (**c**) WT Bleo, (**d**) R_213_G Bleo, scale bar = 100 μm. (**e**) Radial alveolar counts in WT and R_213_G mice following IP PBS or bleomycin exposure, * *p* < 0.05 vs. WT PBS by two-way ANOVA, *n* = 5–8. (**f**) Mean linear intercept in WT and R_213_G mice following IP PBS or bleomycin exposure, scale bar = 200 μm * *p* < 0.05 vs. WT PBS by two-way ANOVA; # *p* < 0.05 vs. WT Bleo by two-way ANOVA *n* = 7–8.

Bleomycin causes impaired alveolar development in WT mice [16]. We performed morphometric assessment of WT mice and mice expressing the R_213_G SNP exposed to either bleomycin or PBS at 22 days with radial alveolar counts (RAC) and mean linear intercept (MLI). As we have previously shown, in WT mice, RAC decreased and MLI increased after bleomycin exposure (Figure 6e,f). Mice expressing the R_213_G SNP displayed no evidence of alveolar injury following bleomycin (Figure 6e,f). 

### 3.7. Bleomycin Only Decreased VEGFR2 and GTPCH-1 Levels in WT Mice

**Figure 7:**
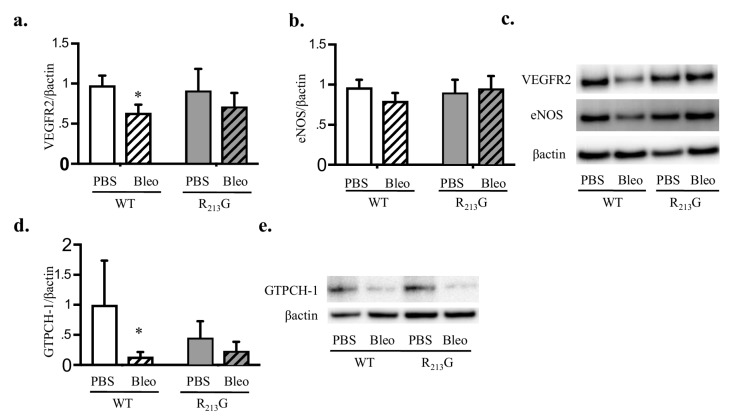
Bleomycin decreased vascular endothelial growth factor receptor 2 (VEGFR2) and guanosine triphosphate cyclohydrolase-1 (GTPCH-1) levels in WT mice. VEGFR2, endothelial nitric oxide synthase (eNOS) and GTPCH-1 protein content in the lung were evaluated by western blot analysis in WT and R_213_G mice at 22 days of age after exposure to PBS (10 μL for 9 doses) or Bleomycin (3 units/kg/dose dissolved in 10 μL of PBS for 9 doses). 25 μg lung protein was loaded onto the gels. Representative blots are shown along with optical density normalized to β-actin and expressed relative to WT control mice. (**a**) VEGFR2 (**b**) eNOS (**c**) Representative blots for VEGFR2 and eNOS (**d**) GTPCH-1, (**e**) Representative blot for GTPCH-1 * *p* < 0.05 vs. WT PBS, by two-way ANOVA, *n* = 5–6 for all groups.

We evaluated VEGFR2 and eNOS protein expression as evidence for impaired vascular endothelial growth factor/nitric oxide (VEGF/NO) signaling. We previously reported these two proteins were decreased in neonatal WT mice treated with bleomycin and mice lacking SOD3 at baseline [16]. We also tested GTPCH-1 expression as it is the rate-limiting enzyme responsible for tetrahydrobiopterin synthesis, a necessary cofactor for eNOS activity. GTPCH-1 is decreased in the setting of impaired SOD3 expression [30,31]. At baseline, VEGFR2 was not significantly different between R_213_G mice and WT mice (Figure 7a,c). We recapitulated our finding that bleomycin decreased lung VEGFR2 in WT mice (Figure 7a,c). In R_213_G mice, bleomycin did not decrease VEGFR2 compared to control (Figure 7a,c). There was a trend toward decreased eNOS in the bleomycin treated WT mice but no overall significant change between strain or exposure (Figure 7b,c). Bleomycin decreased GTPCH-1 in WT mice (Figure 7d,e). In the R_213_G mice GTPCH-1 expression did not change after bleomycin (Figure 7d,e). 

## 4. Discussion

The human R_213_G SOD3 SNP decreases the ability for SOD3 to electrostatically tether to the extracellular matrix and vasculature, resulting in the release of SOD3 from the tissue (lung and vasculature) into the extracellular fluids. This SNP imparts a divergent disease susceptibility, with adult humans at higher risk for ischemic heart disease but lower risk for COPD and asthma [13,15]. Adult mice also exhibit parallel differences in disease susceptibility, demonstrating PH at baseline and aggravated hypoxia induced PH, but protection from LPS and bleomycin lung injury [7,12,14]. In this study, we test the impact of SOD3 redistribution on neonatal lung development and response to bleomycin induced BPD and PH. We report that immature R_213_G mice demonstrate a redistribution of active SOD3 from the lung into the circulation, similar to adult counterparts with consistent activity after bleomycin. The 22 day old R_213_G mice exhibit pulmonary vascular remodeling and PH under control conditions that persist but do not worsen in the setting of bleomycin induced lung injury. Despite the baseline vascular abnormalities, the R_213_G mice have normal alveolar development and are protected against alveolar injury in bleomycin induced BPD. Our study highlights the need to consider the compartmental redox-regulated signaling pathways that account for the discrepancy between vascular and alveolar development attributed to the altered distribution of SOD3. These findings have broad implications for a better understanding of the role for SOD3 in the neonatal lung and consideration of novel approaches to harness this information for more effective therapies.

SOD3 expression and activity are developmentally regulated, thus it was first necessary to evaluate SOD3 distribution in the immature R_213_G mouse. We observe that the distribution of SOD3 was similar in 22 day old and adult R_213_G mice, with decreased lung SOD3 and increased serum SOD3 expression and SOD activity [7,14]. Our next important finding is that the redistribution of SOD3 due to the R_213_G SNP impairs pulmonary vascular development and leads to PH in the developing lung but does not disrupt alveolar development. These findings are consistent with adult mice expressing the R_213_G SNP, who exhibit PH at baseline, however display normal pulmonary function, with normal airway reactivity and pulmonary mechanics [7,12,14]. In contrast, total body lack of SOD3 results in PH at Denver altitude, and impairs both pulmonary vascular development as well as alveolarization [16]. This suggests that the loss of bound SOD3 worsens vascular development but the presence of alveolar SOD3, despite low lung levels, is sufficient to allow normal alveolar development. Based on the known function of SOD3 to reduce superoxide, generate hydrogen peroxide and preserve nitric oxide (NO) bioactivity, we speculate that the loss of bound SOD3 due to the R_213_G SNP alters the local redox state in the vessel wall and contributes to abnormal vascular development and PH at baseline [32]. This could occur through a loss of NO bioactivity due to the inactivation of NO. Alternatively, insufficient vascular bound SOD3 may disrupt redox sensitive growth factors or cell adhesion molecules necessary for angiogenesis, either through increased local superoxide or insufficient vascular hydrogen peroxide [33,34,35,36,37]. As alveolar development is not impaired in the R_213_G mice, bound SOD3 must disrupt separate mechanisms than complete deficiency, where abnormal alveolar and vascular development occur in parallel. These findings add to the literature by demonstrating that in the neonatal lung, SOD3 localization as well as production is critical. This lays a foundation for further investigation to determine which redox regulated signaling pathways are mediated by the loss of vascular SOD3 during development.

We also show that the redistribution of SOD3 due to the R_213_G SNP prevents bleomycin induced lung injury and does not worsen the underlying pulmonary vascular abnormalities or PH. After bleomycin exposure, serum SOD3 and SOD activity in R_213_G mice remains elevated compared to WT mice. The mechanism for protection in R_213_G mice from bleomycin induced lung injury is currently unclear, however we speculate the elevated SOD3 in extracellular fluids attenuates bleomycin induced alveolar injury, pulmonary vascular development and PH by decreasing bleomycin induced oxidative stress. This is supported by work from our lab demonstrating the R_213_G adult mice exhibit decreased alveolar oxidative stress after intratracheal LPS, and preserved reduced glutathione in the lung after intratracheal bleomycin [7,14]. Alternatively, increased serum SOD3 may modulate lung inflammation, as adult R_213_G mice develop less alveolar inflammation following intratracheal LPS and show enhanced resolution of bleomycin induced inflammation [7,14]. These findings are in contrast to immature SOD3 knock out mice, who exhibit both aggravated BPD and worsened pulmonary vascular impairments and PH after bleomycin exposure [16]. These findings emphasize the need to understand the etiology of lung injury and PH in individuals, which may vary depending on the stimulus and individual genetic factors. Collectively, these results illustrate that while the total loss of SOD3 worsens both BPD and PH, the redistribution of SOD3 has a discordant effect on vascular vs. alveolar abnormalities in the setting of experimental BPD and PH.

Abnormalities in the VEGF/NO signaling pathway are widely implicated in the pathogenesis of BPD and PH [16,38,39,40]. VEGFR2 and eNOS expression are decreased in SOD3 knockout mice at baseline and in WT mice after bleomycin injury [16]. GTPCH-1 is the rate limiting enzyme for BH4, an essential cofactor for eNOS, and its deficiency leads to eNOS uncoupling. GTPCH-1 is decreased in ovine neonatal PH and is rescued by recombinant SOD [41,42,43]. Additionally, smooth muscle cell (SMC) selective knock down of SOD3 decreases GTPCH-1 in hypoxia induced PH [30]. We found bleomycin significantly decreases lung VEGFR2 and GTPCH-1 in WT mice, suggesting the impairment in VEGF/NO signaling is multifactorial, with both loss of VEGF signaling and eNOS uncoupling in the setting of vascular and alveolar injury. It is possible that altered NO signaling contributes to vascular abnormalities at baseline in the R_213_G mice as our data support an overall trend towards lower levels of lung GTPCH-1 compared to WT. Following bleomycin, there were no changes in lung VEGFR2, eNOS or GTPCH-1 in the R_213_G mice. This provides further support that the VEGF/NO signaling pathway is not solely responsible for the pathologic changes observed in bleomycin induced lung injury [24,26,44,45,46,47,48].

These data raise interesting observations regarding the relationship between alveolar and pulmonary vascular development. The findings in the R_213_G mice indicate that impaired vascular development does not always occur in parallel with impaired alveolar development. While numerous studies of neonatal BPD and PH demonstrate interdependent angiogenesis and alveolar development [38,39,40,49], other studies, such as a murine postnatal growth restriction and an ovine model of hypoxia induced neonatal PH, show normal alveolar growth despite neonatal vascular impairments and PH, consistent with this current study [50,51]. In addition, other models of neonatal lung injury are characterized by increased angiogenesis and highlight the importance of developmental stage as well as stimulus of injury [52,53]. To further illustrate that different mechanisms can drive alveolar and vascular impairments in the developing lung, multiple therapeutic strategies investigated in the bleomycin model of BPD and PH, including Rho-kinase inhibition, inhaled NO, and serotonin antagonism only ameliorate pulmonary vascular remodeling and PH, but fail to prevent bleomycin-induced alveolar damage [24,26,44,45,46,47,48]. These studies and ours highlight the complexity of normal pulmonary development and have important implications in understanding the risk factors and approach to treatment for at risk preterm infants. 

A limitation of this study is that alveolar and vascular development are dynamic processes and only a single time point during the late alveolar stage was evaluated in this study. It is possible that we are missing subtle abnormalities in alveolarization at earlier time points in the R_213_G mice that have resolved with compensatory alveolar growth by 22 days of life, as is seen in some preterm infants with BPD who demonstrate improved airway abnormalities over time [54,55]. Sex differences are increasingly recognized as important in disease susceptibility, and have been demonstrated in neonatal hyperoxic lung injury as well as bleomycin induced lung injury in aged adult mice but not young adult mice [56,57]. While our preliminary analysis does not show a signal for any differences between sex at baseline or after exposure, we do not have a sufficient number of mice in each group to conclusively determine sex differences. In addition, while we did not find any effect of the R_213_G SNP on three key antioxidant enzymes, it is possible that a more comprehensive evaluation of the lung expression profile would identify a compensatory response to low lung SOD3 content in these mice. Additionally, due to technical challenges in neonatal mice, we did not measure bronchial alveolar lavage fluid (BALF) SOD3, though our prior studies in adult mice demonstrated elevated SOD3 in both serum and BALF in R_213_G mice at baseline and with bleomycin.

## 5. Conclusions

We conclude that a change in the distribution of SOD3 due to the R_213_G SNP leads to pulmonary vascular remodeling and PH at baseline, but protects against experimental neonatal lung injury. This has important therapeutic implications, as an improved understanding of where as well as how SOD3 is protective may lead to the development of more specifically targeted antioxidant therapies for the prevention and treatment of BPD and PH.
